# Temperature-responsive mixed-mode column for the modulation of multiple interactions

**DOI:** 10.1038/s41598-022-08475-8

**Published:** 2022-03-15

**Authors:** Kenichi Nagase, Kosuke Matsumoto, Hideko Kanazawa

**Affiliations:** Faculty of Pharmacy, Keio University, 1-5-30 Shibakoen, Minato, Tokyo, 105-8512 Japan

**Keywords:** Analytical chemistry, Green chemistry

## Abstract

In this study, mixed-mode chromatography columns have been investigated using multiple analyte interactions. A mixed-mode chromatography column was developed using poly(*N*-isopropylacrylamide) (PNIPAAm) brush-modified silica beads and poly(3-acrylamidopropyl trimethylammonium chloride) (PAPTAC) brush-modified silica beads. PNIPAAm brush-modified silica beads and PAPTAC brush-modified silica beads were prepared by atom transfer radical polymerization. The beads were then packed into a stainless-steel column in arbitrary compositions. The elution studies evaluated the column performance on hydrophobic, electrostatic, and therapeutic drug samples using steroids, adenosine nucleotide, and antiepileptic drugs as analytes, respectively. Steroids exhibited an increased retention time when the column temperature was increased. The retention of adenosine nucleotides increased with the increasing composition of the PAPTAC-modified beads in the column. The antiepileptic drugs were separated using the prepared mixed-mode columns. An effective separation of antiepileptic drugs was observed on a 10:1 PNIPAAm:PAPTAC column because the balance between the hydrophobic and electrostatic interactions with antiepileptic drugs was optimized for the bead composition. Oligonucleotides were also separated using mixed-mode columns through multiple hydrophobic and electrostatic interactions. These results demonstrate that the developed mixed-mode column can modulate multiple hydrophobic and electrostatic interactions by changing the column temperature and composition of the packed PNIPAAm and PAPTAC beads.

## Introduction

Chromatography is widely used for the separation and purification of compounds. Various types of chromatography, such as reverse-phase and ion-exchange chromatography, have been investigated depending on the properties of the feed compounds^1–4^. For example, reversed-phase chromatography uses a hydrophobic group to separate hydrophobic compounds (i.e., an octadecyl group), and a modified stationary phase aids in retaining the compounds through hydrophobic interactions^3^. Ion-exchange chromatography employs an ionic-group-modified stationary phase to retain the compounds through electrostatic interactions and to separate the ionic compounds^4^. These hydrophobic or electrostatic interactions are mainly modulated by changing the composition of the mobile phase, such as the addition of an organic solvent and electrolytes into the mobile phase, respectively.

Temperature-responsive chromatography has been investigated as a new type of chromatography because the properties of the stationary phase can be modulated by changing the temperature using an aqueous isocratic mobile phase^5–7^. Poly(*N*-isopropylacrylamide) (PNIPAAm) is a thermoresponsive polymer that exhibits temperature-dependent hydrophilic and hydrophobic attributes across a phase transition temperature of 32 °C^8–10^. Owing to its thermoresponsive properties, PNIPAAm has been used in various applications, such as drug and gene delivery systems^11–16^, biosensor and bioimaging systems^17–22^, nano-actuators^23–27^, cell separation systems^28–34^, and cell culture substrates^35–40^. In a previous study, a PNIPAAm-modified stationary phase was used in a chromatography system because of its hydrophobicity, which changes with temperature^41–45^. The hydrophobic interaction between the stationary phase and analytes can be modulated by changing the column temperature.

To separate ionic compounds using temperature-responsive chromatography, temperature-responsive ion-exchange chromatography has been investigated using thermoresponsive ionic copolymers prepared by the copolymerization of NIPAAm and ionic monomers^46–48^. Chromatography can separate ionic compounds through electrostatic interactions between thermoresponsive ionic polymers and analytes. The electrostatic interactions with analytes can be modulated by changing the composition of the ionic monomer in the PNIPAAm copolymer. However, an excessive incorporation of the ionic monomers into the PNIPAAm copolymer reduces the thermoresponsive properties of the PNIPAAm copolymer^49,50^. Thus, electrostatic solid interactions cannot be utilized in thermoresponsive ion-exchange chromatography.

By contrast, mixed-mode chromatography columns have been investigated for separating analytes with multiple interactions^51^. A mixed-mode chromatography column was developed by packing two types of beads in the same column, namely, hydrophobic group immobilized silica beads and ionic group immobilized silica beads. Analyte interacted with the packed beads through multiple hydrophobic and electrostatic interactions, and the interactions can be modulated by changing the beads composition. If the concept of a mixed mode chromatography is applied to temperature-responsive chromatography, effective separation could be performed through temperature-modulated multiple interactions.

**Figure 1:**
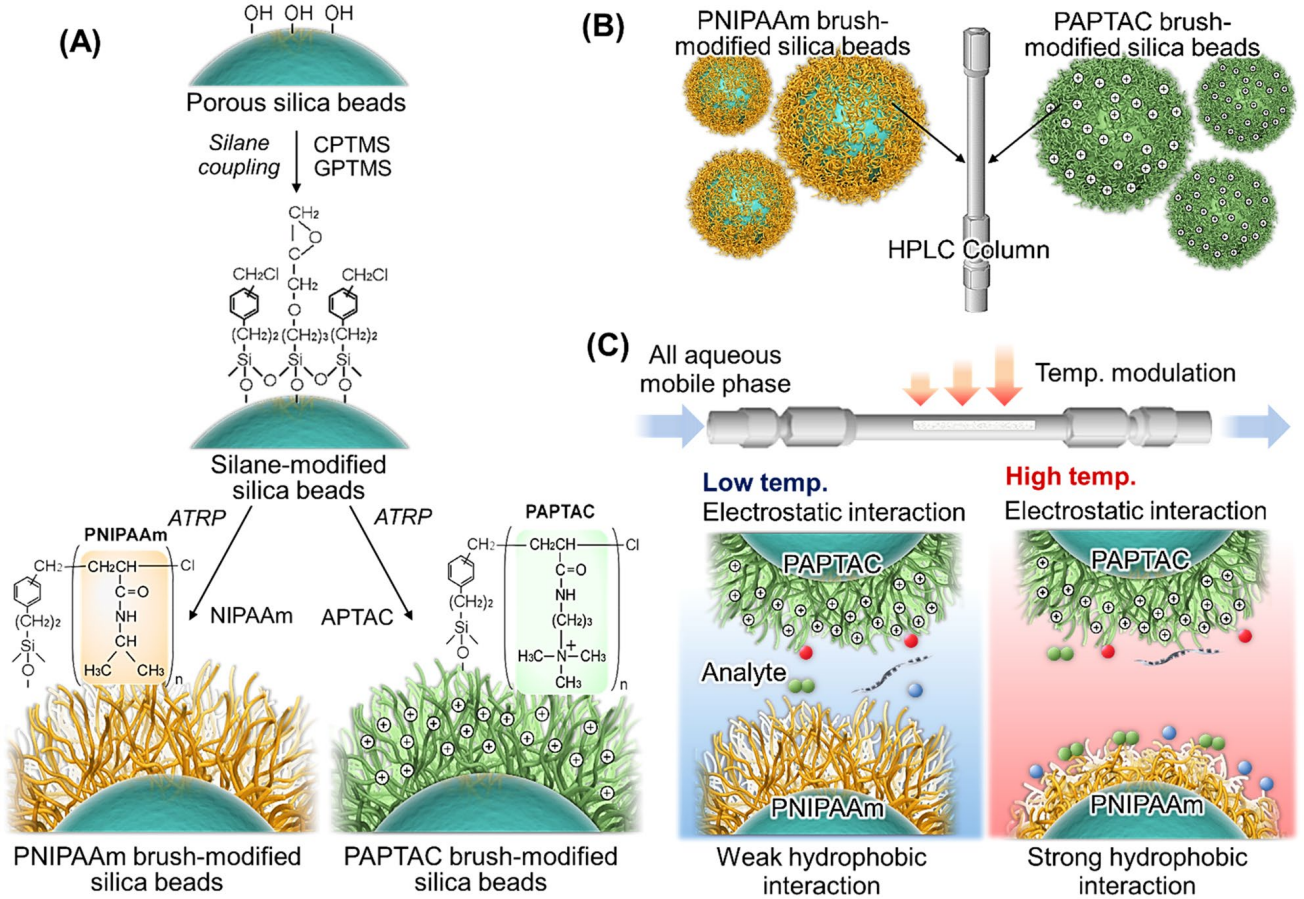
Temperature-responsive mixed-mode column using thermoresponsive-polymer-modified beads and cationic-polymer-modified beads. Schematic illustration of the (**A**) preparation of PNIPAAm (left) and PAPTAC (right) brush-modified beads, (**B**) preparation of the mixed-mode column by packing two types of beads, and (**C**) multiple types of interactions with the analyte. (Figures were drawn Microsoft PowerPoint 2019 Version 2112).

In this study, we developed a temperature-responsive mixed-mode chromatography column using PNIPAAm brush-modified beads and a strong cationic polymer poly(3-acrylamidopropyl) trimethylammonium chloride (PAPTAC) brush-modified beads (Fig. 1). The performance of the bead-packed columns with various packing compositions was investigated by observing the elution behavior of hydrophobic, electrostatic, and therapeutic drug samples represented by steroids, adenosine nucleotides, antiepileptic drugs, and oligonucleotides, respectively.

## Results and discussion

### Characterization of the prepared beads

PNIPAAm and PAPTAC brush-modified silica beads were prepared by a silane coupling reaction and subsequent atom transfer radical polymerization (ATRP) of NIPAAm and APTAC (Fig. 1A). In this study, glycidyloxypropyl trimethoxysilane (GPTMS) was mixed with ((Chloromethyl)phenylethyl)trimethoxysilane (CPTMS) to dilute the initiator’s density, as slightly diluted polymer brush-modified stationary phases exhibit relatively sharp peaks in chromatograms^52^.

The prepared beads were characterized by using the carbon, hydrogen, and nitrogen (CHN) elemental analysis, attenuated total reflection Fourier-transform infrared spectroscopy (ATR/FT–IR), zeta potential observation, and scanning electron microscopy (SEM).

The silica beads were characterized by elemental analysis at each reaction step (Table 1). A larger carbon composition was observed in the silane-layer-modified silica beads than the unmodified silica beads. The results indicated that the silane coupling reaction was successfully performed under the conditions used in the study. The amount of immobilized silane layer composed of CPTMS and GPTMS was 2.12 μmol/m^2^. This value is relatively small compared to the beads solely modified with CPTMS, as previously reported (~ 5.0 μmol/m^2^)^53,54^. This outcome may be due to the molecular size of GPTMS, which is slightly larger than that of CPTMS, thus lowering the immobilized density of the silane layer.

**Table 1:** Characterization of the silane layer and polymer-modified beads. The carbon composition of the beads was determined through elemental analysis (n = 3). %C_(calcd)_ was calculated as the percentage of the molecular weight of carbon in the silane layer and polymer. The amount of layered silane and polymer on silica beads was estimated using the measured carbon composition.

Code	Carbon composition (%)	%C_(calcd)_	Immobilized silane (μmol/m^2^)	Grafted polymer (mg/m^2^)
Unmodified silica beads	0.25 ± 0.04			
Silane-layer-modified silica (CPTMS:GPTMS = 75:25)	2.26 ± 0.03	57.2	2.12	
PNIPAAm-modified silica beads	17.2 ± 0.02	63.7		3.21
PAPTAC-modified silica beads	9.67 ± 0.02	61.5		1.43

The PNIPAAm- and PAPTAC-brush-modified silica beads exhibited a higher carbon composition than the silane-layer-modified silica beads. This result indicated that PNIPAAm and PAPTAC successfully modified the silica beads through ATRP under the studied reaction conditions. The modified PNIPAAm and PAPTAC were 3.21 μmol/m^2^ and 1.43 μmol/m^2^, respectively. These values indicated that a relatively large polymer was modified on the silica beads through the ATRP. A larger amount of PNIPAAm was modified on silica beads compared with PAPTAC due to the higher concentration of NIPAAm monomer compared with that of APTAC in the ATRP. Previous reports indicate that the amount of modified polymer on silica beads increased with increasing monomer concentration in ATRP^55,56^. In the present study, the monomer concentrations of NIPAAm and APTAC were 1000 mM and 300 mM, respectively. The difference in monomer concentration significantly influenced the quantity of modified polymer on the silica beads.

**Figure 2:**
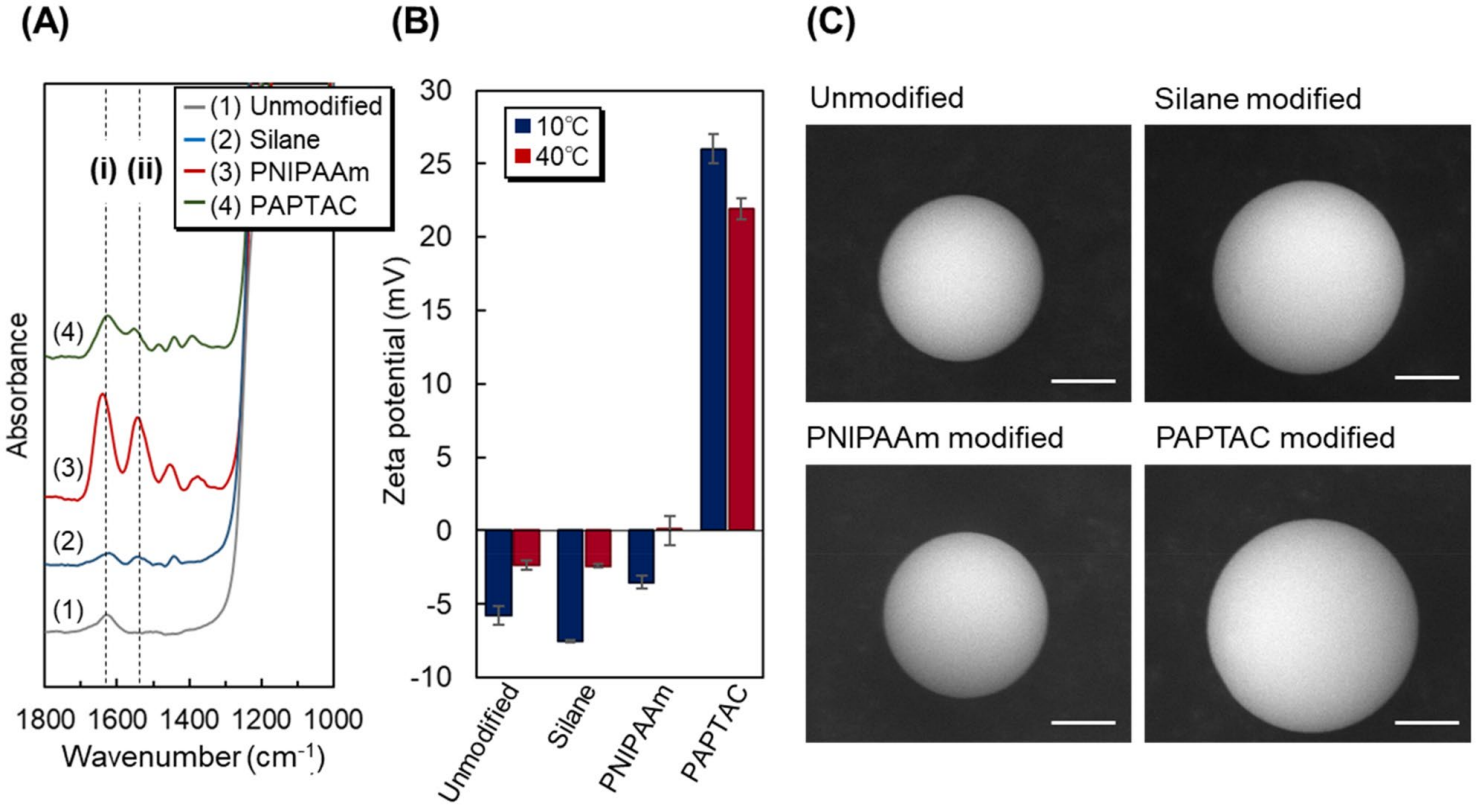
Characterizations of the prepared beads. (**A**) FT–IR spectra of the prepared beads. The dashed lines, (i) and (ii), indicate the peaks attributed to the C=O stretching and N–H bending vibrations, respectively. (**B**) Zeta potential of the prepared silica beads (n = 3). (**C**) SEM images of the prepared beads where the scale bars = 2 μm. (Figures were drawn Microsoft PowerPoint 2019 Version 2112).

The prepared beads were characterized by observing the spectrum of ATR/FT–IR (Fig. 2A). The PNIPAAm- and PAPTAC-modified silica beads exhibited two peaks at 1550 and 1645 cm^−1^ in the spectrum. By contrast, no peaks were observed in the spectrum of unmodified silica beads and silane-layer-modified beads. The results also indicated that PNIPAAm and PAPTAC were successfully modified on silica beads because the two peaks at 1550 and 1645 cm^−1^ were attributed to the amide bonds of PNIPAAm and PAPTAC.

The zeta potentials of the prepared beads at each reaction step were investigated (Fig. 2B). PAPTAC brush-modified silica beads have a cationic group and consequently exhibited a higher zeta potential than other beads. The results indicated that the PAPTAC brush-modified silica beads have strong cationic properties.

The SEM micrographs of the prepared beads at each reaction step were performed (Fig. 2C and Supplementary Fig. S1). Similar spherical shapes were observed in each SEM image, indicating that the silane coupling reaction did not deform the silica beads. Additionally, bead aggregation was not observed after the ATRP of NIPAAm and APTAC, indicating that the polymerization reaction was controlled.

### Elution behavior of analytes from the mixed-mode column

A mixed solvent composed of water:methanol:2-propanol (1:1:1 ratio) was prepared as a suspension of the column packing. The prepared PNIPAAm- and PAPTAC-brush-modified silica beads were suspended in the mixed solvent at weight ratios of 1:0, 20:1, and 10:1. The bead suspension was packed into a stainless-steel column (4.6 mm inner diameter × 50 mm length). The elution behavior of various analytes in the prepared columns was observed.

**Figure 3:**
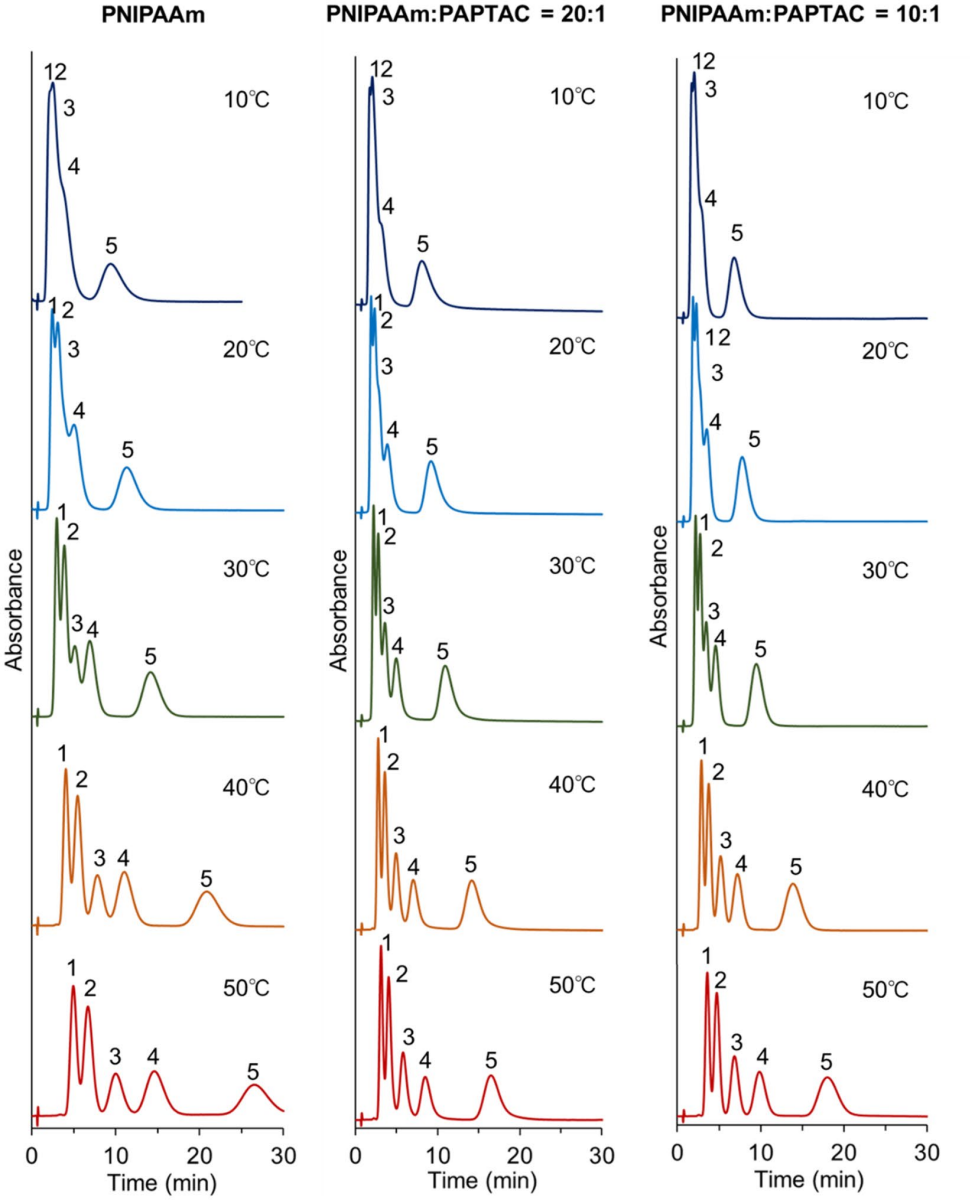
Chromatograms of hydrophobic steroids using prepared mixed-mode columns. The mobile phase was pure water. The flow rate of the mobile phase was 1.0 mL/min, and detection was measured at a wavelength of 260 nm. Peak 1 = hydrocortisone, peak 2 = prednisolone, peak 3 = dexamethasone, peak 4 = hydrocortisone acetate, and peak 5 = testosterone. (Figures were drawn Microsoft PowerPoint 2019 Version 2112).

The elution behavior of the hydrophobic steroids (Supplementary Table S1) was observed to investigate the hydrophobic interaction between the prepared bead-packed columns and analytes (Fig. 3). In all columns, the steroids were eluted in ascending order of hydrophobicity (i.e., hydrocortisone, prednisolone, dexamethasone, hydrocortisone acetate, and testosterone). The results indicated that the steroids were retained on the column through hydrophobic interactions. In addition, the retention time of the steroids increased with increasing column temperature because of the thermoresponsive hydrophobicity change of the PNIPAAm brush-modified beads. PNIPAAm brushes on silica beads in columns were dehydrated and became hydrophobic with increasing temperature, leading to enhanced hydrophobic interactions between PNIPAAm and the steroids and an elongation of the retention time of steroids. These results indicate that hydrophobic interactions between the packed beads in the column and the analyte can be modulated by changing the column temperature.

A previous report on temperature-responsive chromatography using mass spectroscopy as detector indicated that separated analytes maintained their structure without decomposition through column separation^57^.

**Figure 4:**
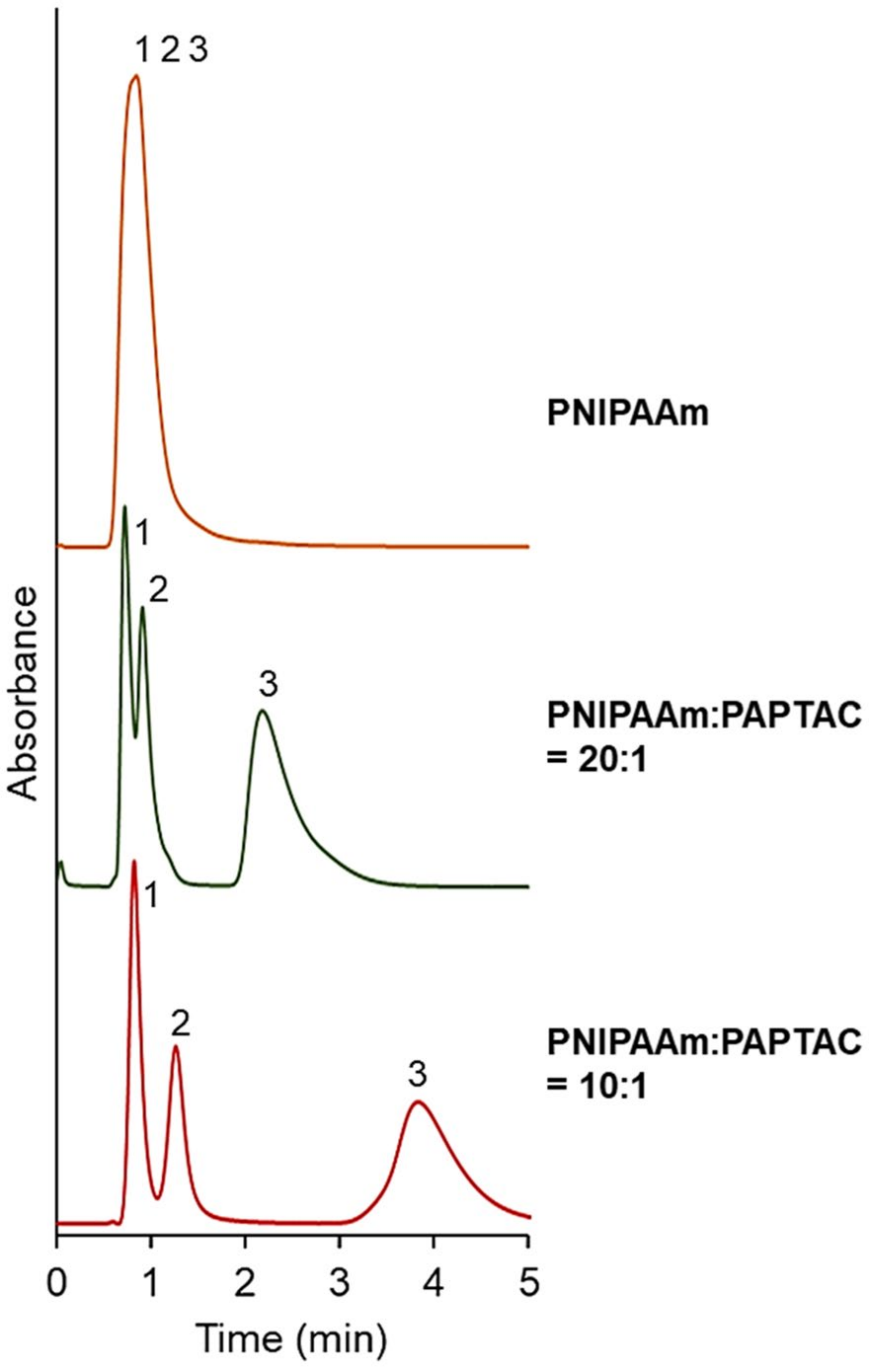
Chromatograms of adenosine nucleotides using prepared mixed-mode columns. The mobile phase is 33.3 mmol/L phosphate buffer solution (pH = 7.0). The flow rate of the mobile phase is 1.0 mL/min, and detection was measured at a wavelength of 260 nm. The column temperature is 40 °C. Peak 1 = AMP, peak 2 = ADP, and peak 3 = ATP. (Figures were drawn Microsoft PowerPoint 2019 Version 2112).

To investigate the electrostatic interaction between the bead-packed column and analyte, the elution behavior of adenosine nucleotides from the prepared column was observed (Fig. 4). The elution behavior of each adenosine nucleotide was also observed (Supplementary Fig. S2). The properties of the adenosine nucleotides are summarized in Supplementary Table S2. Adenosine nucleotides were eluted as one peak on the column packed with PNIPAAm-modified beads. By contrast, the PNIPAAm:PAPTAC = 20:1 and 10:1 columns exhibited three peaks. These results indicated that these adenosine nucleotides were retained in the mixed-mode columns through electrostatic interaction between PAPTAC on the beads in the column and adenosine nucleotides. Adenosine nucleotides eluted in the order of adenosine monophosphate (AMP), adenosine diphosphate (ADP), and adenosine triphosphate (ATP), because the electrostatic interaction between PAPTAC and adenosine nucleotides increased with an increase in the number of phosphoric acids of adenosine nucleotides. In addition, a longer retention time of adenosine nucleotides was observed on the PNIPAAm:PAPTAC = 10:1 column compared to that of the PNIPAAm:PAPTAC = 20:1 column. This outcome may be due to the larger composition of PAPTAC-modified beads in the PNIPAAm:PAPTAC = 10:1 column compared to that of the PNIPAAm:PAPTAC = 20:1 column, leading to strong electrostatic interaction with adenosine nucleotides. The results indicated that the electrostatic interaction with the analyte could be modulated by changing the composition of the PAPTAC-modified beads in the column.

**Figure 5:**
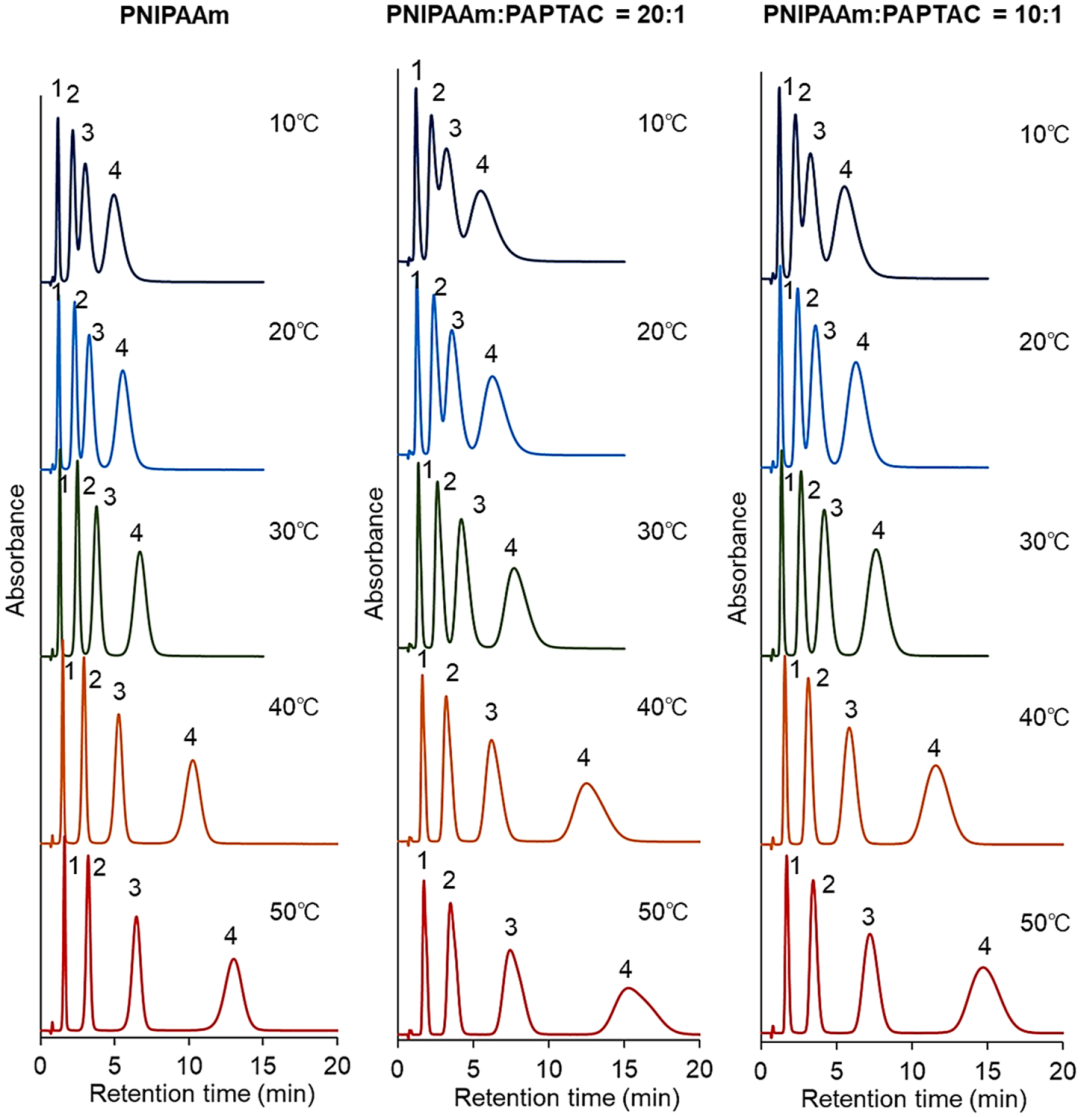
Chromatograms of the antiepileptic drugs on the prepared mixed-mode columns. The mobile phase is 10 mM CH_3_COONH_4_ buffer solution (pH 6.8) with a flow rate of 1.0 mL/min, and detection was measured at a wavelength of 260 nm. Peak 1 = zonisamide, peak 2 = carbamazepine, peak 3 = nitrazepam, and peak 4 = clonazepam. (Figures were drawn Microsoft PowerPoint 2019 Version 2112).

The elution behavior of the antiepileptic drug from the prepared mixed-mode column was observed to investigate the availability of the prepared column for therapeutic drug monitoring (Fig. 5). Antiepileptic drugs require therapeutic drug monitoring to determine the drug concentration in human serum. If the prepared mixed-mode columns can measure the antiepileptic drug concentration, the columns can be used for therapeutic drug monitoring. The properties of antiepileptic drugs are summarized in Supplementary Table S3. In all the prepared columns, the retention time of antiepileptic drugs increased with increasing temperature. The results indicated that antiepileptic drugs were retained on the column through hydrophobic interactions with PNIPAAm. PNIPAAm on the packed beads in the column dehydrate and become hydrophobic with increasing column temperature, resulting in enhanced hydrophobic interactions and an elongation of the retention time of analytes. PNIPAAm:PAPTAC = 20:1 and PNIPAAm:PAPTAC = 10:1 columns exhibited longer retention times of antiepileptic drugs than those of the PNIPAAm beads packed column alone. The results indicated that the electrostatic interaction between antiepileptic drugs and PAPTAC also contributed to the retention of antiepileptic drugs. The PNIPAAm:PAPTAC = 20:1 column exhibited the longest retention time for antiepileptic drugs. The results indicated an appropriate balance between hydrophobic and electrostatic interactions for the retention of antiepileptic drugs. The reproducibility of the analysis was verified through repeated measurements of the elution behavior of antiepileptic drugs (Supplementary Fig. S3 and Supplementary Table S4). Relatively similar retention profiles of antiepileptic drugs were observed. In addition, the relative standard deviation of the retention times with repeated measurements was relatively small. These results indicate that the method is reproducible.

**Figure 6:**
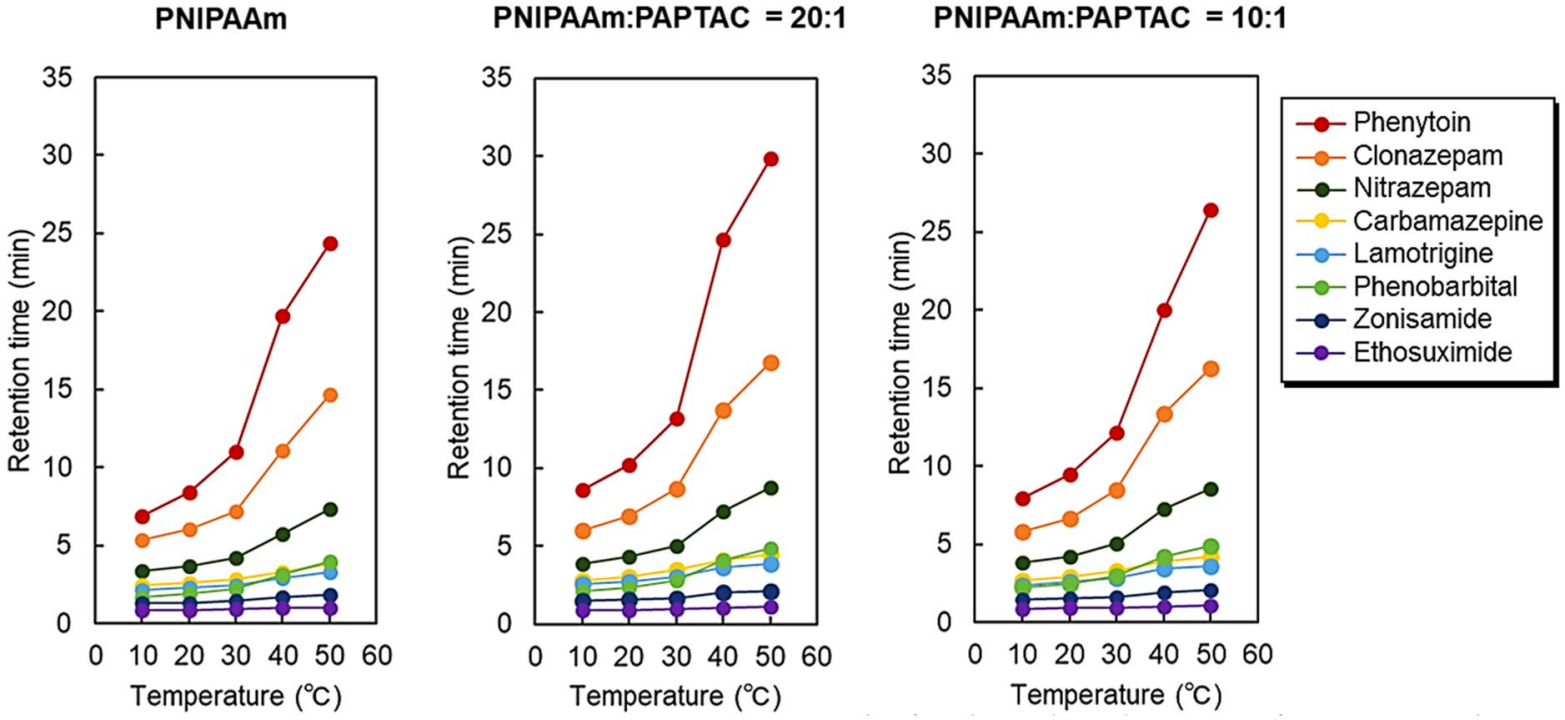
Retention times of the antiepileptic drugs on the prepared mixed-mode columns. (Figures were drawn Microsoft PowerPoint 2019 Version 2112).

**Figure 7:**
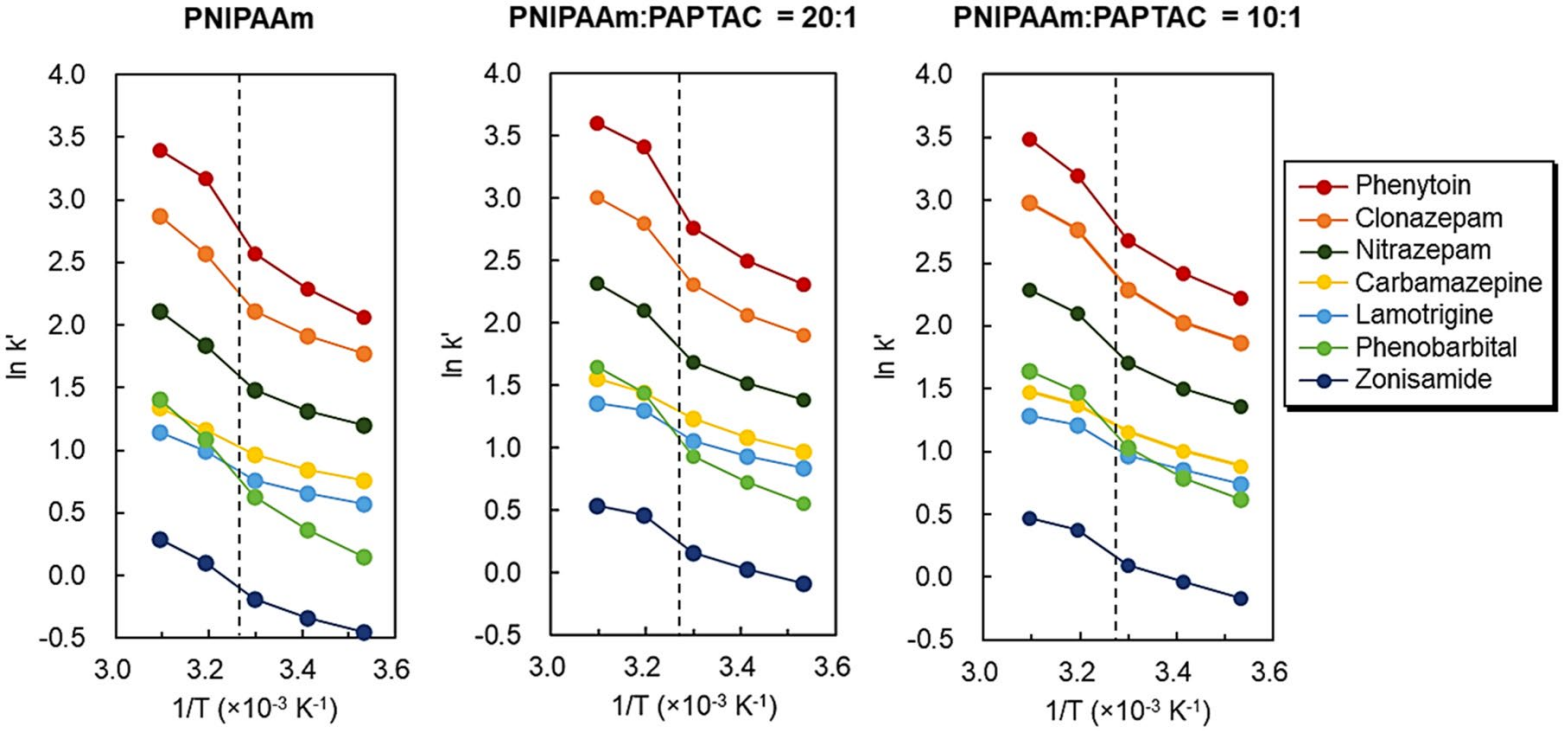
van’t Hoff plots of the antiepileptic drugs on the prepared mixed-mode columns. The dashed line indicates the phase transition temperature of PNIPAAm. (Figures were drawn Microsoft PowerPoint 2019 Version 2112).

Further investigation of the temperature-dependent retention profiles of the antiepileptic drugs was observed (Supplementary Fig. S4). The temperature-dependent changes in the retention time of antiepileptic drugs on the prepared mixed-mode columns are shown in Fig. 6. In addition, van’t Hoff plots for analyzing analyte retention with temperature were obtained (Fig. 7). A significant increase in retention time was observed between 30 and 40 °C in all the columns. This behavior may be due to the PNIPAAm dehydration, which proceeded significantly at the phase transition temperature of PNIPAAm (32 °C). Thus, hydrophobic interactions between PNIPAAm and antiepileptic drugs were significantly enhanced, leading to a significant increase in retention time between 30 and 40 °C. The van’t Hoff plots exhibited a negative slope, indicating that the analyte retention increased with increasing temperature. In addition, the slope changed near the phase transition temperature of PNIPAAm, which is also attributed to the enhanced retention of analytes on PNIPAAm as dehydration proceeded.

**Figure 8:**
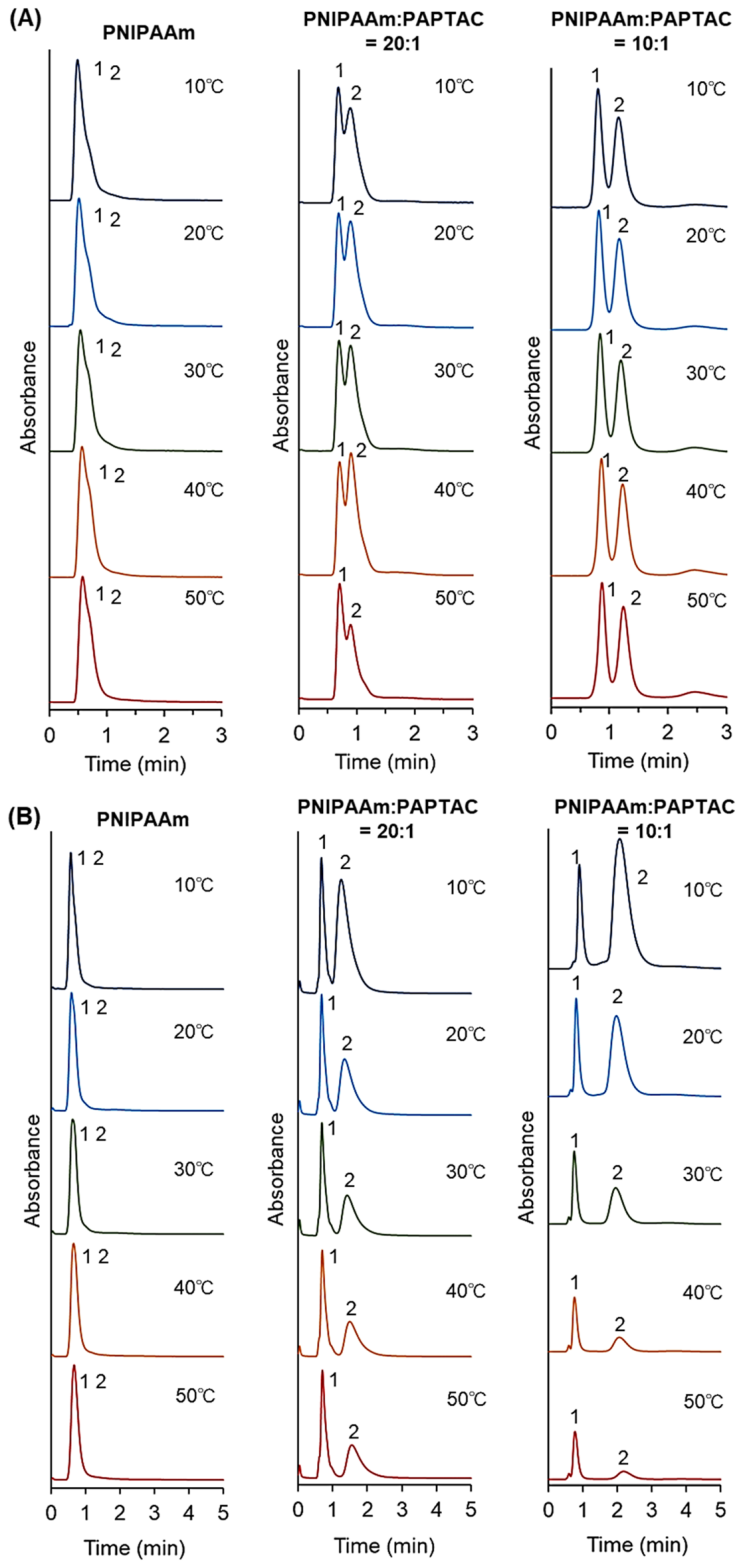
Chromatograms of oligonucleotides on prepared mixed-mode columns. (**A**) Mixture of d(T)_5_ and d(T)_6_. The mobile phase is 66.7 mM phosphate buffer solution (pH = 7.0) with a flow rate of 1.0 mL/min. Detection was measured at a wavelength of 260 nm. Peak 1 = d(T)_5_ and peak 2 = d(T)_6_. (**B**) Mixture of d(T)_5_ and d(T)_10_. The mobile phase is 66.7 mM phosphate buffer solution (pH = 7.0) + 100 mM NaCl with a flow rate of 1.0 mL/min. Detection was measured at a wavelength of 260 nm. Peak 1 = d(T)_5_ and peak 2 = d(T)_10_. (Figures were drawn Microsoft PowerPoint 2019 Version 2112).

The elution behavior of the oligonucleotides from the prepared mixed-mode column was observed (Fig. 8). The elution behavior of each type of oligonucleotide was also observed (Supplementary Fig. S5), and the temperature-dependent peak area change was determined (Supplementary Fig. S6). The properties of the oligonucleotides used are summarized in Supplementary Table S5. Oligonucleotide therapeutics have attracted attention as effective therapies. Thus, the separation of oligonucleotides would be helpful in the purification and quality control of oligonucleotide therapeutics. In the elution behavior of a mixture of d(T)_5_ and d(T)_6_, two types of oligonucleotides were eluted at one peak on the PNIPAAm brush-modified beads packed column at all column temperatures. The results indicated that hydrophobic interactions between PNIPAAm and oligonucleotides were not adequate for the retention of oligonucleotides.

On the contrary, on the PNIPAAm:PAPTAC = 20:1 column, the retention times of these oligonucleotides increased, and the peak tops were slightly separated. On the PNIPAAm:PAPTAC = 10:1 column, the oligonucleotides were separated. These results indicated that the electrostatic interaction between PAPTAC and oligonucleotides enhanced the retention of oligonucleotides. The PNIPAAm:PAPTAC = 10:1 column can separate oligonucleotides with different lengths of a single base, 5mer, and 6mer. The elution behavior of oligonucleotides d(T)_5_ and d(T)_10_ from the prepared columns was also observed. On the column packed with PNIPAAm-modified beads, d(T)_5_ and d(T)_10_ eluted as one peak at all temperatures because the hydrophobic interaction between PNIPAAm and oligonucleotides was not adequate for retention on the column even at relatively long base oligonucleotides. The PNIPAAm:PAPTAC = 20:1 column and PNIPAAm:PAPTAC = 10:1 column can separate a mixture of oligonucleotides with baseline separation. Longer retention times of oligonucleotides were exhibited on the PNIPAAm:PAPTAC = 10:1 column than on the PNIPAAm:PAPTAC = 20:1 column. This outcome may be due to the electrostatic interaction between PAPTAC and oligonucleotides, which increased with increasing composition of the packed PAPTAC in the column. As the PNIPAAm:PAPTAC = 10:1 column temperature increases, the elution of d(T)_10_ oligonucleotides decreases, as displayed by the decreased peak areas measured in the chromatogram (Supplementary Fig. S6C). This result could be due to the oligonucleotide adsorption at an elevated column temperature proceeding through multiple hydrophobic and electrostatic interactions. The temperature-dependent adsorption and desorption of analytes is a specific property of temperature-responsive ion-exchange chromatography. Previous studies reported the enhancement of protein adsorption onto a thermoresponsive ionic copolymer-modified stationary phase at elevated column temperatures. This was found to be due to multiple hydrophobic and electrostatic interactions between proteins and the thermoresponsive ionic polymer^58–60^. In the case of oligonucleotides with long bases, such as d(T)_10_, the oligonucleotides interact strongly with PAPTAC on the beads in PNIPAAm:PAPTAC = 10:1 and PNIPAAm:PAPTAC = 20:1 columns. In addition, the hydrophobic interaction between the oligonucleotides and PNIPAAm increased with increasing temperature, leading to the adsorption of oligonucleotides on the stationary phase at elevated column temperatures. Thus, our results indicate that a low temperature is suitable for the high-recovery separation of oligonucleotides with base lengths similar to d(T)_10_.

These results demonstrate that the developed mixed-mode column can modulate multiple hydrophobic and electrostatic interactions simply by changing the column temperature and composition of the packed PNIPAAm and PAPTAC beads. The column can separate antiepileptic drugs and oligonucleotides with all aqueous mobile phases. Thus, the developed mixed-mode columns would be useful for drug monitoring in pharmaceutical therapy and the purification of oligonucleotide therapeutics.

## Conclusions

A mixed-mode temperature-responsive chromatography column was developed by preparing PNIPAAm brush-modified beads and PAPTAC brush-modified beads through ATRP and then packing these beads in arbitrary compositions. PNIPAAm and PAPTAC were successfully modified on silica beads through ATRP, confirmed by elemental analysis, FT-IR, zeta potential measurement, and SEM. The prepared beads were packed into a stainless-steel column in compositions of PNIPAAm, PNIPAAm:PAPTAC = 20:1, and PNIPAAm:PAPTAC = 10:1. The elution behavior of the steroids from the prepared mixed-mode columns was observed. The retention time of the steroids increased with increasing column temperature, indicating that the hydrophobic interaction between the analytes and PNIPAAm increased with increasing temperature, which was attributed to the dehydration of PNIPAAm. Adenosine nucleotide retention increased with increasing packed PAPTAC composition in the columns, which was attributed to electrostatic interaction between adenosine nucleotides, and PAPTAC increased with the composition of the packed PAPTAC-modified beads in the column. Antiepileptic drugs were separated by increasing the column temperature through multiple hydrophobic and electrostatic interactions. The highest separation efficiency of antiepileptic drugs was observed in the PNIPAAm:PAPTAC = 10:1 column, indicating that an optimal balance between hydrophobic and electrostatic interactions can be obtained at that composition of the beads. Oligonucleotides were separated using PNIPAAm:PAPTAC = 20:1 and PNIPAAm:PAPTAC = 10:1 columns. Further, the PNIPAAm column could not separate oligonucleotides, indicating that multiple hydrophobic and electrostatic interactions are required to separate oligonucleotides. These results indicate that the developed mixed-mode column can modulate multiple hydrophobic and electrostatic interactions by simply varying the column temperature and composition of the packed PNIPAAm and PAPTAC beads. The column can separate antiepileptic drugs and oligonucleotides with all aqueous mobile phases. Thus, the developed mixed-mode columns would be useful for drug monitoring in pharmaceutical therapy, and the purification of oligonucleotide therapeutics.

## Methods

### Preparation of polymer brush-modified beads

Packing materials of the temperature-responsive mixed-mode column were prepared by a silane coupling reaction for initiator modification and by the subsequent ATRP for polymer modification (Fig. 1). All materials used in the experiments are summarized in the Supplementary Information. Silica beads (10.0 g) were washed with hydrochloric acid at 90 °C for 3 h^50^. The beads were filtered, rinsed with pure water, and dried in a vacuum drying oven (DP200, Yamato, Tokyo) at 150 °C for 16 h, following which the silica beads (5.0 g) were humidified in 500 mL flasks (humidity = 60%) at 25 °C for 4 h^61^. CPTMS (2.89 mL, 11.8 mmol) and GPTMS (0.845 mL, 3.83 mmol) were dissolved in toluene (286 mL)^52,62,63^. The prepared reaction solution was poured into the silica beads in the flask and then the silane coupling reaction was performed at 25 °C for 16 h with continuous stirring. Then, the silica beads were filtered, rinsed with acetone, and dried at 110 °C for 3 h^34^.

PNIPAAm modification was performed following the ATRP. PNIPAAm (4.86 g, 42.9 mmol) was solved in 42.8 mL 2-propanol in a 100 mL flask, and the monomer solution was deoxygenated under flowing argon gas for 30 min^59^. Then, CuCl (84.41 mg, 0.8526 mmol), CuCl_2_ (11.34 mg, 0.08434 mmol), and Me_6_TREN (220.6 mg, 0.9575 mmol) were dissolved into the solution under argon gas atmosphere^34^. The flask was then sealed, and the solution was stirred using a magnetic stirrer at 25 °C for 10 min. The reaction solution and initiator-immobilized silica beads (1.0 g) in a 50 mL glass vessel were placed in a glove bag. The oxygen in the glove bag was removed by repeated vacuuming, and argon gas was flowed thrice^59^. The reaction solution was poured into the silica beads in the glass vessel, which was then sealed. ATRP was performed with continuous shaking of the bead suspension at 25 °C for 16 h using a shaker (SN-M40S, Nissin, Tokyo). After the ATRP, the beads were poured into two centrifuge tubes (50 mL), and acetone (20 mL) was poured into the tubes. The beads were washed by sonication for 30 min to remove the monomers, followed by centrifugation at 1000 rpm for 3 min^64^. The supernatant was removed, and a mixture of 50 mM EDTA aqueous solution and methanol (1:1) was added to the centrifuged tubes. The beads were washed by sonication for 30 min to remove the ATRP catalyst, followed by centrifugation at 1000 rpm for 3 min^34^. The bead suspensions were then filtered, rinsed with pure water and acetone, and dried at 50 °C for 3 h^62^.

For the PAPTAC modification on silica beads, APTAC (3.10 g, 0.015 mol) was dissolved in 50 mL of a 2-propanol:water (1:1) mixture solvent. The monomer solution was passed to the inhibitor remover column to remove the polymerization inhibitor in APTAC^49,65^. Then, the same procedure was followed as described for the modification of PNIPAAm.

### Characterization of polymer brush-modified beads

The prepared beads were characterized by CHN elemental analysis, FT-IR, zeta potential measurement, and SEM.

The carbon composition of the prepared beads was investigated at each reaction step to determine the amount of silane layer and polymer on the beads^62^. The carbon composition was determined using an elemental analyzer (PE-2400, PerkinElmer, Waltham, MA, USA)^60^. The amount of modified silane layer comprising CPTMS and GPTMS was obtained using Eq. (1) as follows:

1 $$\frac{\%{C}_{S}}{\%{C}_{S}\left(calcd\right) \times \left(1-\%{C}_{S}/\%{C}_{S}(calcd)\right) \times S},$$

where *%C*_*S*_ is the increase in the carbon content of the beads after silane coupling reaction, %*C*_*S*_ (*calcd*) is the calculated carbon percentage of the mixed silane coupling reagents composed of CPTMS and GPTMS (3:1), and *S* is the surface area of the silica beads (130 m^2^/g)^34,59^. The amount of modified PNIPAAm was obtained using Eq. (2) as follows:

2 $$\frac{\%{C}_{N}}{\%{C}_{N}\left(calcd\right)\times \left(1-\%{C}_{N}/\%{C}_{N}(calcd)-\%{C}_{S}/\%{C}_{S}(calcd)\right)\times S},$$

where *%C*_*N*_ is the increase in the carbon content of the beads after ATRP of NIPAAm, and %*C*_*N*_ (*calcd*) is the calculated carbon percentage of NIPAAm^34^. The amount of modified PNIPAAm was obtained using Eq. (3) as follows:

3 $$\frac{\%{C}_{A}}{\%{C}_{A}(calcd)\times \left(1-\%{C}_{A}/\%{C}_{A}(calcd)-\%{C}_{S}/\%{C}_{S}(calcd)\right)\times S},$$

where *%C*_*A*_ is the increase in the carbon content of the beads after ATRP of APTAC, and %*C*_*A*_ (*calcd*) is the calculated carbon percentage of APTAC. The polymer modification of the beads in each reaction step was confirmed by ATR FT–IR (FT/IR-4700, JASCO, Tokyo, Japan)^59^.

The zeta potential of the beads was determined using a zeta potential analyzer (Zetasizer Nano-ZS, Malvern Instruments, Malvern, UK). Bead suspensions (0.5 mg/mL) were prepared in a 5 mmol/L potassium chloride solution for zeta potential measurements^59^. The morphology of the silica beads at each reaction step was observed using SEM (TM4000Plus-II, Hitachi High-tech, Tokyo, Japan)^41^.

### Elution behavior of analytes from mixed-mode column

A mixed-mode column was prepared by combining PNIPAAm and PAPTAC beads and packing them into an empty column (4.6 inner diameter × 50 mm length)^44^. Solely PNIPAAm-modified beads packed column and PNIPAAm-modified beads (0.8 g) were suspended in 20 mL of a 1:1:1 mixed solvent of water:methanol:2-propanol. In the case of the PNIPAAm:PAPTAC = 20:1 column, PNIPAAm-modified beads (0.764 g) and PAPTAC-modified beads (0.0382 g) were suspended in the mixed solvent, while in that of PNIPAAm:PAPTAC = 10:1 column, PNIPAAm-modified beads (0.727 g) and PAPTAC-modified beads (0.0727 g) were suspended in the mixed solvent. The bead suspension was poured into the reservoir of the column packer that was connected to an empty column^44^. Beads were packed to enable a continuous flow of a mixed solvent with a continuous pressure of 350 kg/cm^2^ for 1 h^43^. Then, the column was connected to a high-performance liquid chromatography system (Chromaster, Hitachi High-Tech Science, Tokyo, Japan), which was rinsed with pure water flowing at 1.0 mL/min at 40 °C for 5 h.

To investigate hydrophobic interactions between the prepared stationary phase and the analyte, hydrophobic steroids were used as model analytes. The properties of the steroids are listed in Supplementary Table S1. Each steroid (5 mg) was dissolved in methanol (1 mL), and pure water was added to a total volume of 5 mL. The solution was then filtered using a syringe filter (pore diameter: 0.2 μm). The filtered solution (0.5 mL) was added to pure water (2.5 mL). The prepared solution was used as a steroid sample^59^. Steroid elution from the prepared column was observed at a detection wavelength of 260 nm. The mobile phase was pure water with a flow rate of 1.0 mL/min. The column temperature was controlled using a column oven (Chromaster 6310, Hitachi High-Tech Science, Tokyo, Japan).

An adenosine nucleotide was used as a model sample to investigate the electrostatic interaction between the developed stationary phase and analytes. The properties of the adenosine nucleotides are summarized in Supplementary Table S2. Each adenosine nucleotide (5 mg) was dissolved in 5 mL of 66.7 mM phosphate buffer (pH = 7.0)^59^. The solution was filtered using a syringe filter. AMP (500 μL), ADP (500 μL), and ATP (1500 μL) solutions were mixed, and 2.5 mL of 66.7 mM phosphate buffer (pH = 7.0) was added to the solution^59^. The resultant solution was used as a sample of the adenosine nucleotides. The elution behavior of adenosine nucleotides was observed at a detection wavelength of 260 nm. The mobile phase was 33.3 mM phosphate buffer (pH = 7.0) with a flow rate of 1.0 mL/min.

The elution behavior of antiepileptic drugs from the prepared column was observed to investigate the availability of the prepared column for therapeutic drug monitoring because it is required by pharmaceutical therapy. The properties of antiepileptic drugs are summarized in Supplementary Table S3. For phenobarbital and ethosuximide, each drug was dissolved in 5 mL of 10 mM CH_3_COONH_4_ solution (pH 6.8). In case of other antiepileptic drugs, the drug was dissolved in 2 mL of methanol, and 10 mM CH_3_COONH_4_ solution (pH = 6.8) was added to 5 mL of the solution. The solution was then filtered through a syringe filter, and 500 µL of it was added to 4.5 mL of 10 mM CH_3_COONH_4_ solution (pH = 6.8) to adjust the concentration to 100 µg/mL. An antiepileptic drug solution was used as the sample. The elution behavior of the drug was observed in the prepared column at a detection wavelength of 260 nm. The mobile phase was 10 mM CH_3_COONH_4_ solution (pH = 6.8) with a flow rate of 1.0 mL/min.

To investigate the retention behavior on the column, van’t Hoff plots were obtained for the analytes. The retention factor, *k*′, was obtained using Eq. (4) as follows:

4 $$k^{\prime} = \frac{{t_{R} - t_{0} }}{{t_{0} }},$$

where *t*_*R*_ is the retention time of the analyte and *t*_*0*_ is the retention time of uracil as an initial standard^43^. Oligonucleotides were used as a sample for nucleic acid medicine^66^. The properties of the oligonucleotides are listed in Supplementary Table S5. Oligonucleotides were dissolved in 66.7 mM phosphate buffer solution (pH = 7.0) at a concentration of 49 µmol/L, except for d(T)_6_, which was dissolved at a concentration of 41 µmol/L. The elution behavior of the oligonucleotides was observed at a detection wavelength of 260 nm^67^. The mobile phase was 66.7 mM phosphate buffer solution (pH = 7.0) with a flow rate of 1.0 mL/min for a mixture of d(T)_5_ and d(T)_6_ and was a mixture of 66.7 mM phosphate buffer solution (pH = 7.0) and 100 mM NaCl for a mixture of d(T)_5_ and d(T)_10_.

## Supplementary Information

Supplementary Information.

## Data Availability

The datasets generated during and/or analyzed during the current study are available from the corresponding author on reasonable request.
