# Supplementary material for: Temperature-responsive mixed-mode column for the modulation of multiple interactions
Source: Sci Rep. 2022 Mar 15;12:4434. doi: 10.1038/s41598-022-08475-8 (PMC8924202; doi:10.1038/s41598-022-08475-8)
Supplement: Supplementary file 1 — Supplementary Information. [file 41598_2022_8475_MOESM1_ESM.docx]

Supplementary Information

**Temperature-responsive mixed-mode column for the modulation of multiple interactions**

*Kenichi Nagase*, Kosuke Matsumoto, and Hideko Kanazawa*

Faculty of Pharmacy, Keio University, 1-5-30 Shibakoen, Minato, Tokyo 105-8512, Japan.

*Corresponding author: (Phone) +81-3-5400-1378; (E-mail) nagase-kn@pha.keio.ac.jp

**Materials**

*N-*isopropylacrylamide (NIPAAm) and 3-acrylamidopropyl trimethylammonium chloride (APTAC) were provided by KJ Chemicals (Tokyo, Japan). NIPAAm was purified by recrystallization from *n*-hexane. APTAC was purified by removing the polymerization inhibitor using an inhibitor removal column (Sigma-Aldrich, St Louis, MO, USA). Methanol, hydrochloric acid, acetone, toluene, tris(2-aminoethyl)amine, 2-propanol, copper(I) chloride, copper(II) chloride, ammonium acetate, hydrocortisone, prednisolone, dexamethasone, hydrocortisone acetate, testosterone, acetic acid, phenytoin, clonazepam, nitrazepam, carbamazepine, phenobarbital, and zonisamide were purchased from Fujifilm Wako Chemicals (Osaka, Japan). Tris[2-(dimethylamino)ethyl]amine (Me_6_TREN) was synthesized from tris(2-aminoethyl)amine. Glycidyloxypropyltrimethoxysilane (GPTMS), adenosine monophosphate, adenosine diphosphate, adenosine triphosphate, lamotrigine, and ethosuximide were obtained from Tokyo Chemical Industry (Tokyo, Japan). Oligonucleotides were purchased from Tsukuba Oligo Service (Tsukuba, Japan). ((Chloromethyl)phenylethyl)trimethoxysilane was purchased from Gelest (Morrisville, PA, USA). Silica beads (EP-DF-5 300A(SV)) (diameter: 5 μm; pore diameter: 300 Å; 130 m^2^/g) were provided by AGC Si-Tech (Fukuoka, Japan). Stainless-steel columns (inner diameter: 4.6 mm; column length: 50 mm) were purchased from GL Science (Tokyo, Japan).

**Supplementary Table S1.** Properties of hydrophobic steroids

| Compounds | Structure | Molecular weight | LogP |
| --- | --- | --- | --- |
| Hydrocortisone | 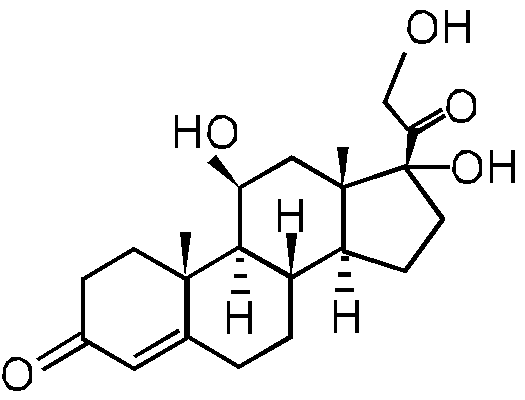 | 362.46 | 1.61 |
| Prednisolone | 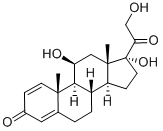 | 360.44 | 1.62 |
| Dexamethasone | 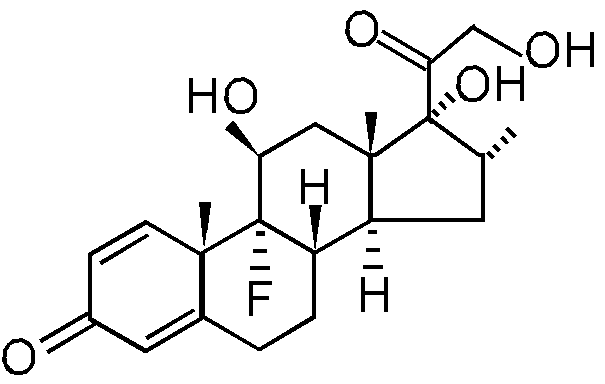 | 392.46 | 1.83 |
| Hydrocortisone acetate | 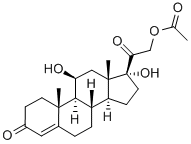 | 404.50 | 2.30 |
| Testosterone | 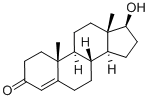 | 288.42 | 3.32 |

**Supplementary Table S2.** Properties of adenosine nucleotides

| Compounds | Structure | Molecular weight | LogP | p*K*_a_ |
| --- | --- | --- | --- | --- |
| AMP | 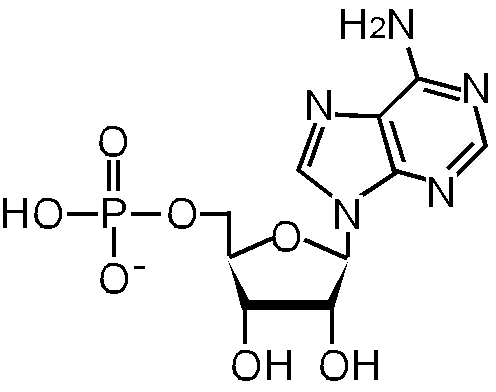 | 347.06 | -3.45 | 3.8 |
| ADP | 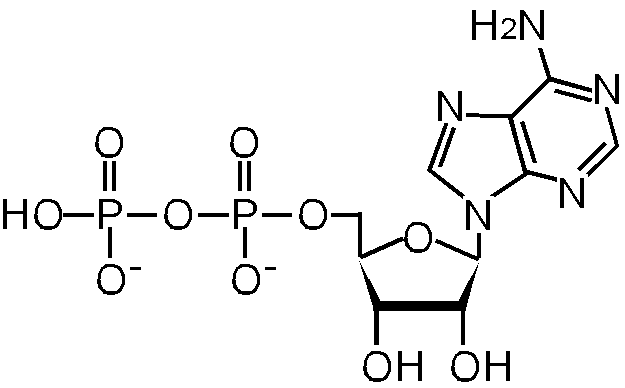 | 427.20 | -4.00 | 3.9 |
| ATP | 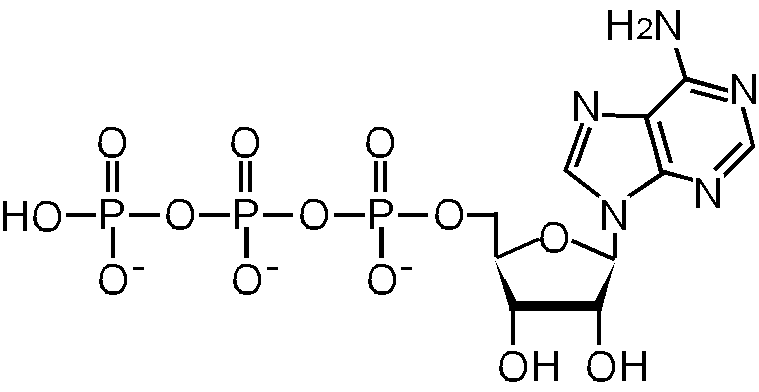 | 507.18 | -4.66 | 4.1 |

**Supplementary Table S3.** Properties of antiepileptic drug

| Compounds | Structure | Molecular weight | Log*P* ^a^ | p*K*_a_ |
| --- | --- | --- | --- | --- |
| Phenytoin | 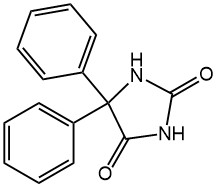 | 252.27 | 2.47 | 8.33 |
| Clonazepam | 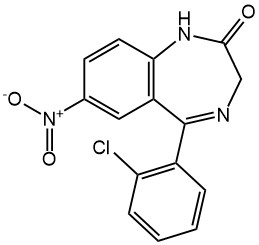 | 315.7 | 2.41 | 1.61 |
| Nitrazepam | 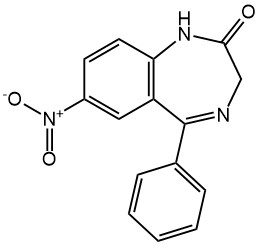 | 281.27 | 2.25 | 2.80 |
| Carbamazepine | 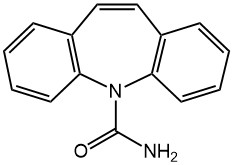 | 236.27 | 2.45 | −3.80, 15.96 |
| Lamotrigine | 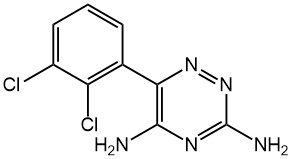 | 256.09 | 2.57 | 5.70 |
| Phenobarbital | 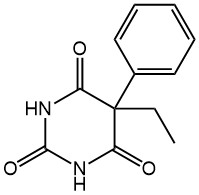 | 232.24 | 1.47 | 7.30 |
| Zonisamide | 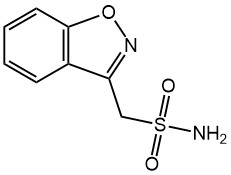 | 212.23 | 0.50 | 9.66 |
| Ethosuximide | 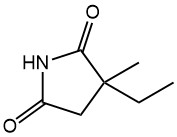 | 141.2 | 0.38 | 9.3 |

**
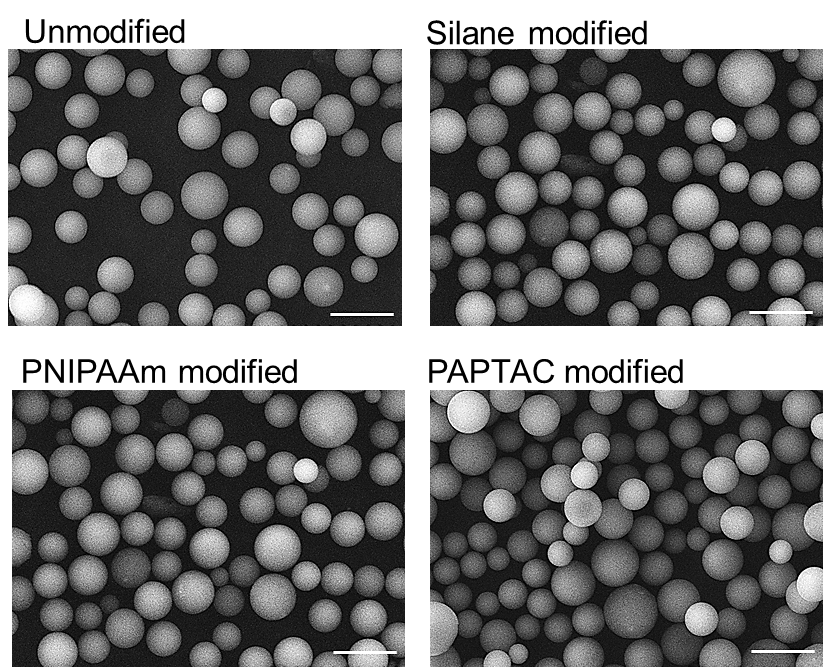
**

**Supplementary Fig. S1.** SEM images of the prepared beads with scale bars equal to 10 μm. (Figures were drawn Microsoft PowerPoint 2019 Version 2112)

**
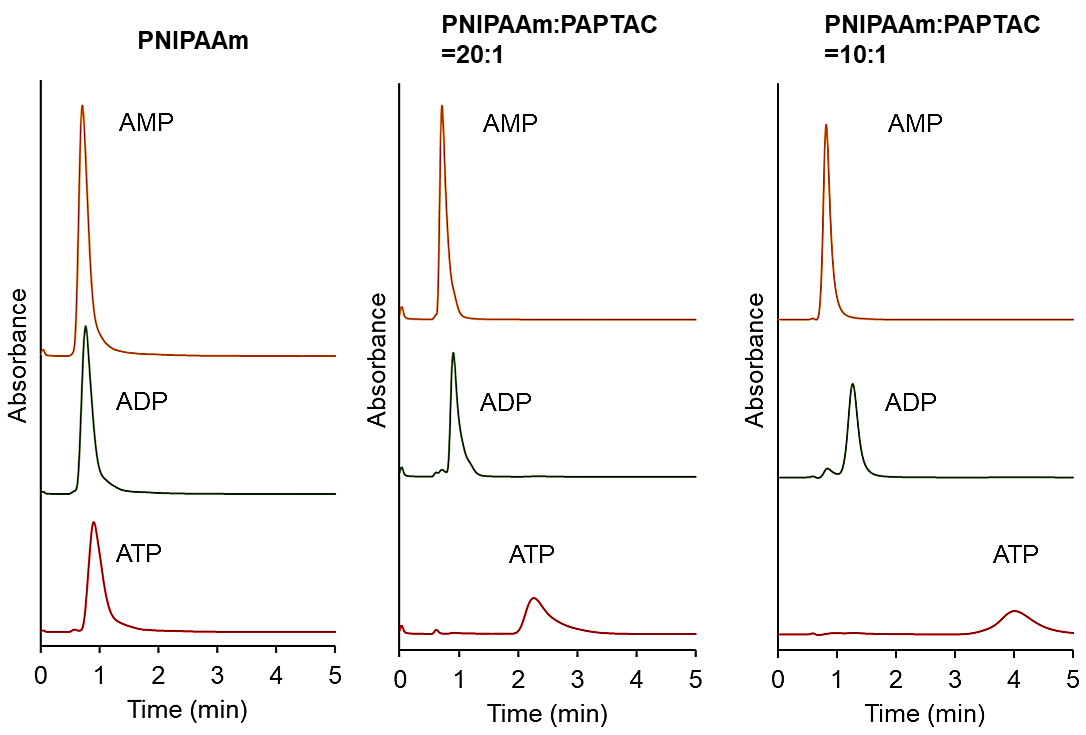
**

**Supplementary Fig. S2.** Chromatograms of adenosine nucleotides using prepared mixed-mode columns at a temperature of 40 °C. The mobile phase is 33.3 mmol/L phosphate buffer solution (pH = 7.0) with a flow rate of 1.0 mL/min. Detection was measured at a wavelength of 260 nm. (Figures were drawn Microsoft PowerPoint 2019 Version 2112)

**
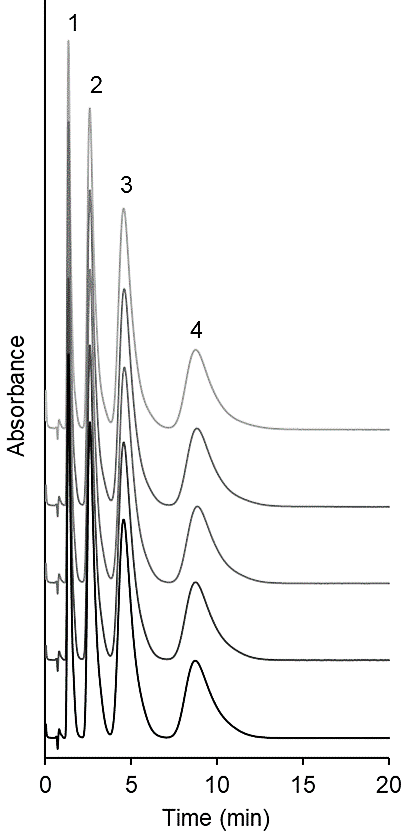
**

**Supplementary Figure S3.** Chromatograms of the antiepileptic drug on the prepared mixed-mode column (PNIPAAm:PAPTAC = 20:1) with five repeated measurements. The mobile phase is 10 mM CH_3_COONH_4_ buffer solution (pH = 6.8) with a flow rate of 1.0 mL/min. The column temperature is 40 °C. Detection was measured at a wavelength of 260 nm. Peak 1 = zonisamide, peak 2 = carbamazepine, peak 3 = nitrazepam, and peak 4 = clonazepam. (Figures were drawn Microsoft PowerPoint 2019 Version 2112)

**Supplementary Table S4.** Retention time of antiepileptic drugs with repeated measurements (n=5)

| Drugs | Retention time (min) ^a)^ | RSD (%) ^b)^ |
| --- | --- | --- |
| Zonisamide | 1.36 | 0.180 |
| Carbamazepine | 2.60 | 0.176 |
| Nitrazepam | 4.58 | 0.385 |
| Clonazepam | 8.78 | 0.520 |

a) The retention times of the drugs were measured at 40 °C using a 10 mM CH_3_COONH_4_ buffer solution (pH = 6.8) as the mobile phase and averaged after five separate measurements. b) The relative standard deviation was obtained by dividing the standard deviation of the retention time by the retention time and multiplying it.

**
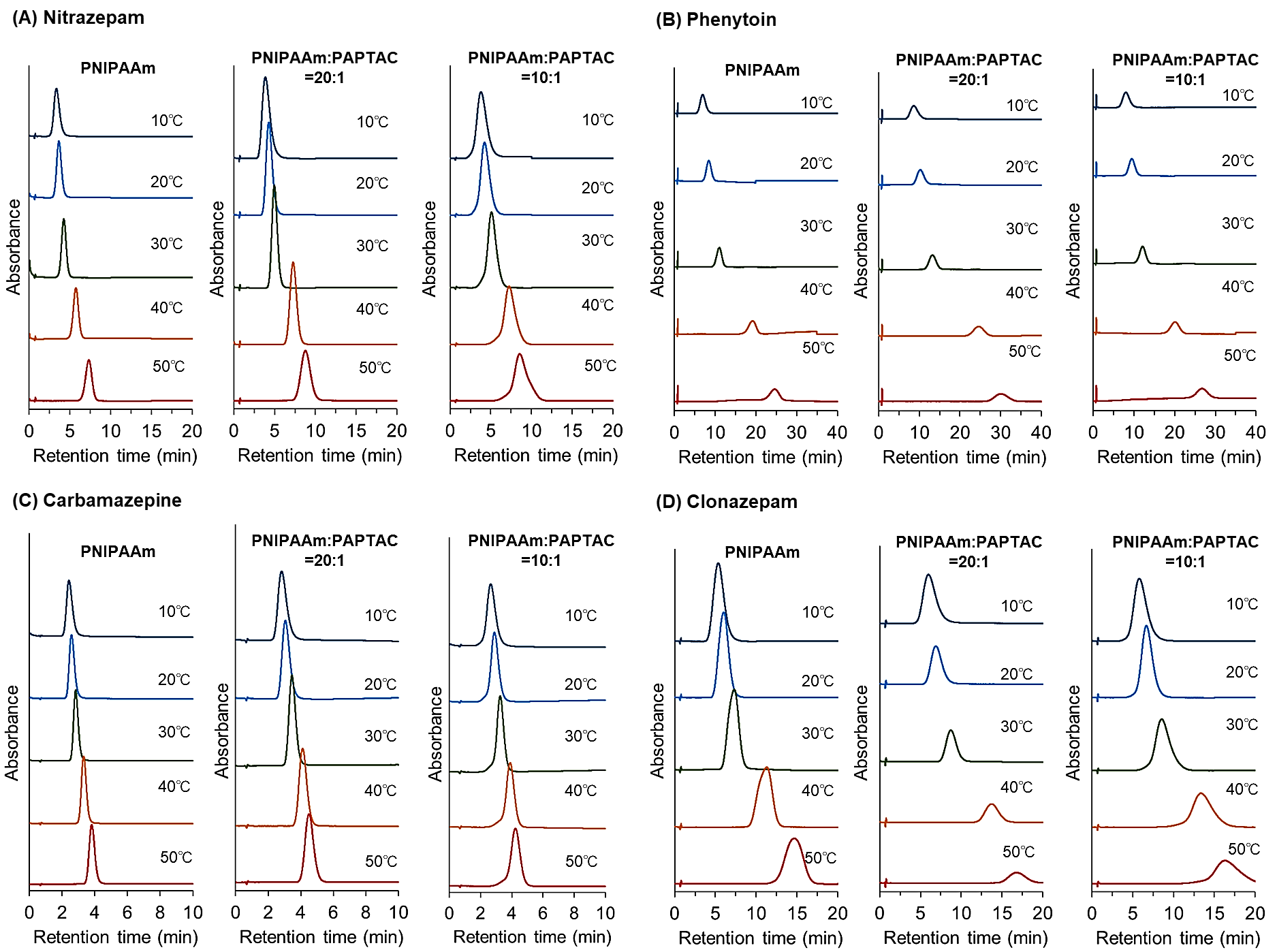
**

**Supplementary Fig. S4.1** Chromatograms of antiepileptic drugs on prepared mixed mode columns with five measurements recorded at various temperatures. The mobile phase is a 10 mM CH_3_COONH_4_ buffer solution (pH 6.8) with a flow rate of 1.0 mL/min. Detection was measured at a wavelength of 260 nm. (A) nitrazepam, (B) phenytoin, (C) carbamazepine, and (D) clonazepam. (Figures were drawn Microsoft PowerPoint 2019 Version 2112)

**
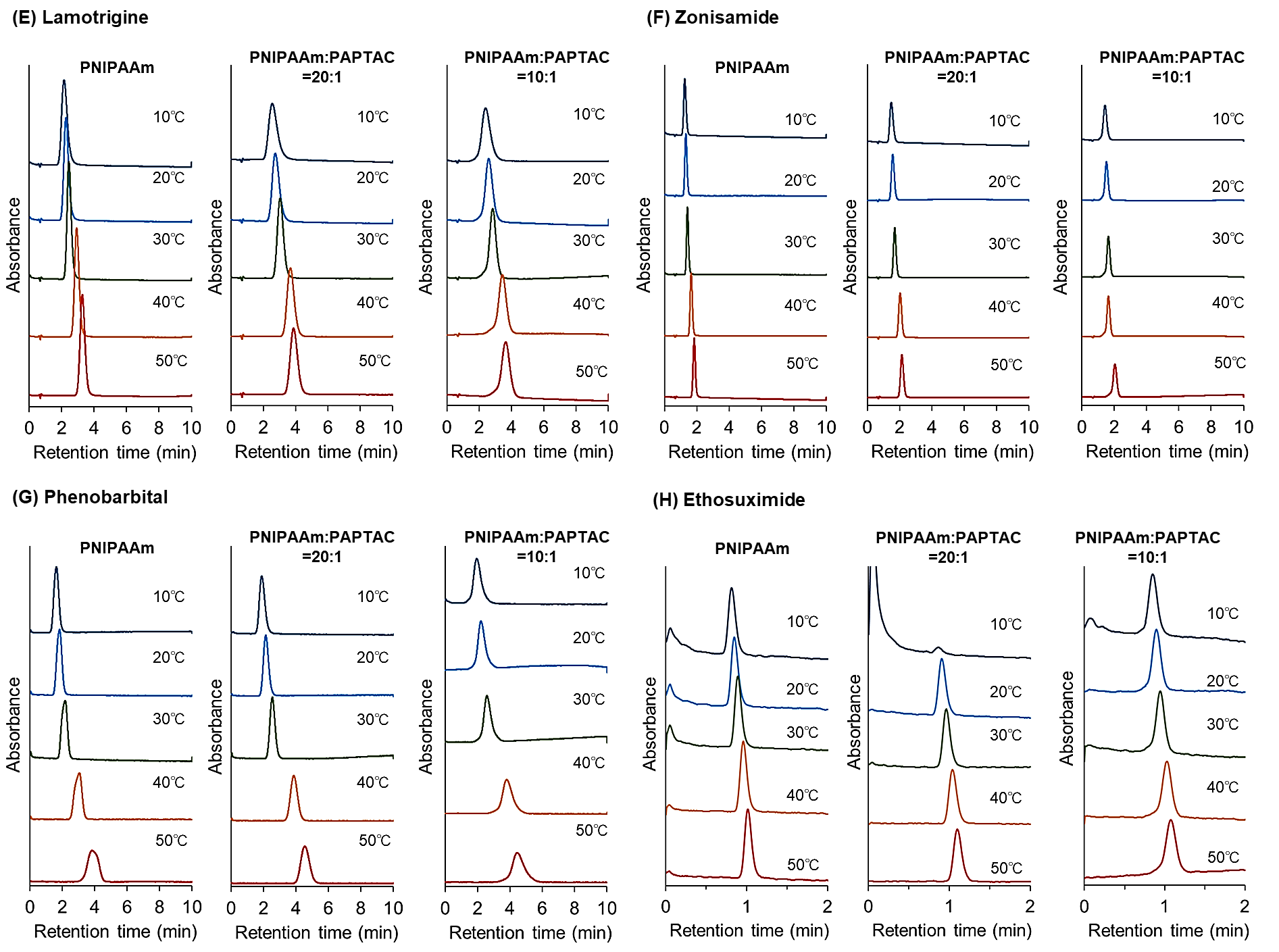
**

**Supplementary Fig. S4.2** Chromatograms of antiepileptic drugs on prepared mixed mode columns with five measurements recorded at various temperatures. The mobile phase is a 10 mM CH_3_COONH_4_ buffer solution (pH 6.8) with a flow rate of 1.0 mL/min. Detection was measured at a wavelength of 260 nm. (E) lamotrigine, (F) zonisamide, (G) phenobarbital, and (H) ethosuximide. (Figures were drawn Microsoft PowerPoint 2019 Version 2112)

**Supplementary Table S5.** Properties of oligonucleotides

| Sample | Sequence | Length |
| --- | --- | --- |
| d(T)_5_ | 5’-d(TTTTT)-3’ | 5mer |
| d(T)_6_ | 5’-d(TTTTTT)-3’ | 6mer |
| d(T)_10_ | 5’-d(TTTTTTTTTT)-3’ | 10mer |

**
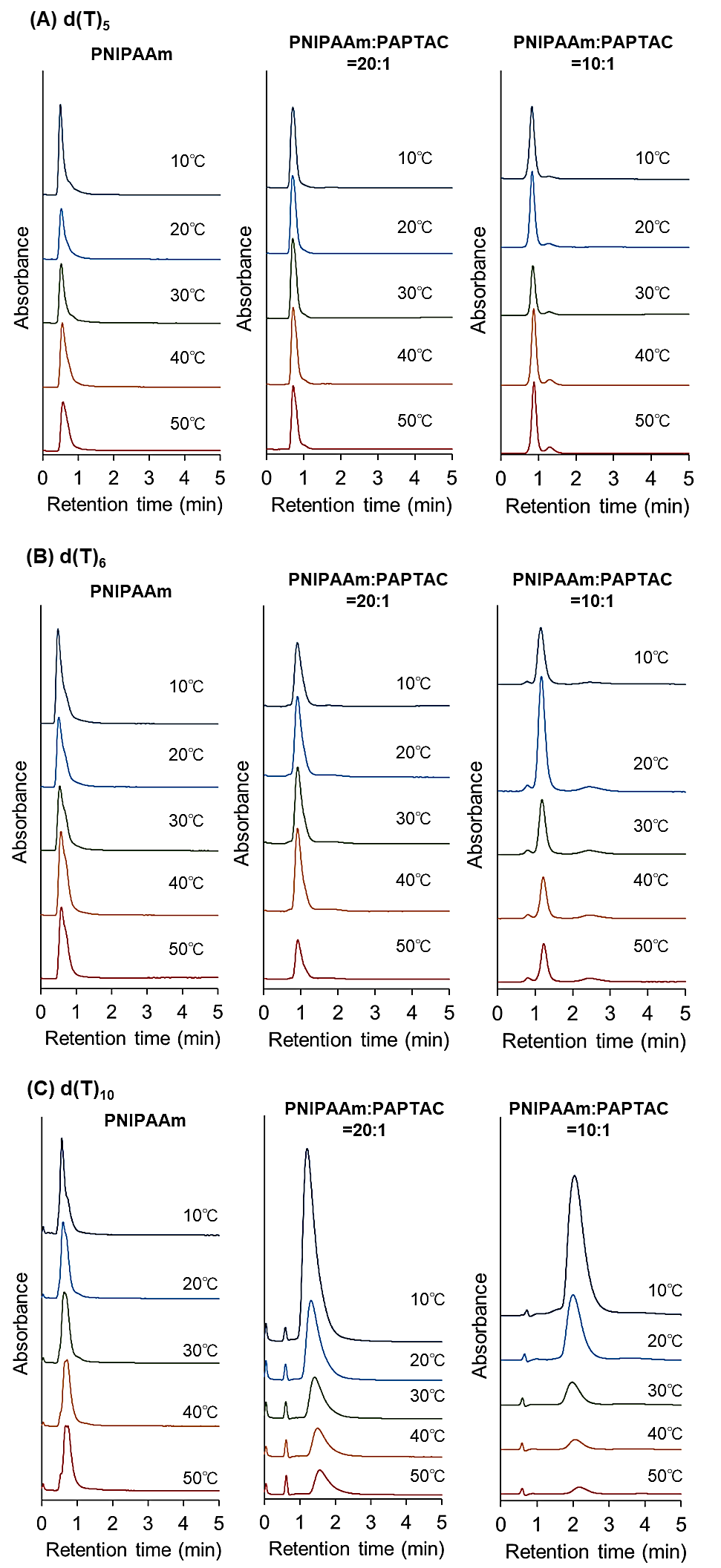
**

**Supplementary Fig. S5** Chromatograms of oligonucleotides on the prepared mixed mode columns. (A) d(T)_5_ using 66.7 mM phosphate buffer solution (pH = 7.0), (B) d(T)_6_ using 66.7 mM phosphate buffer solution (pH = 7.0), and (C) d(T)_10_ using 66.7 mM phosphate buffer solution (pH = 7.0) + 100 mM NaCl. A flow rate of 1.0 mL/min was used. Detection was measured at a wavelength of 260 nm. (Figures were drawn Microsoft PowerPoint 2019 Version 2112)


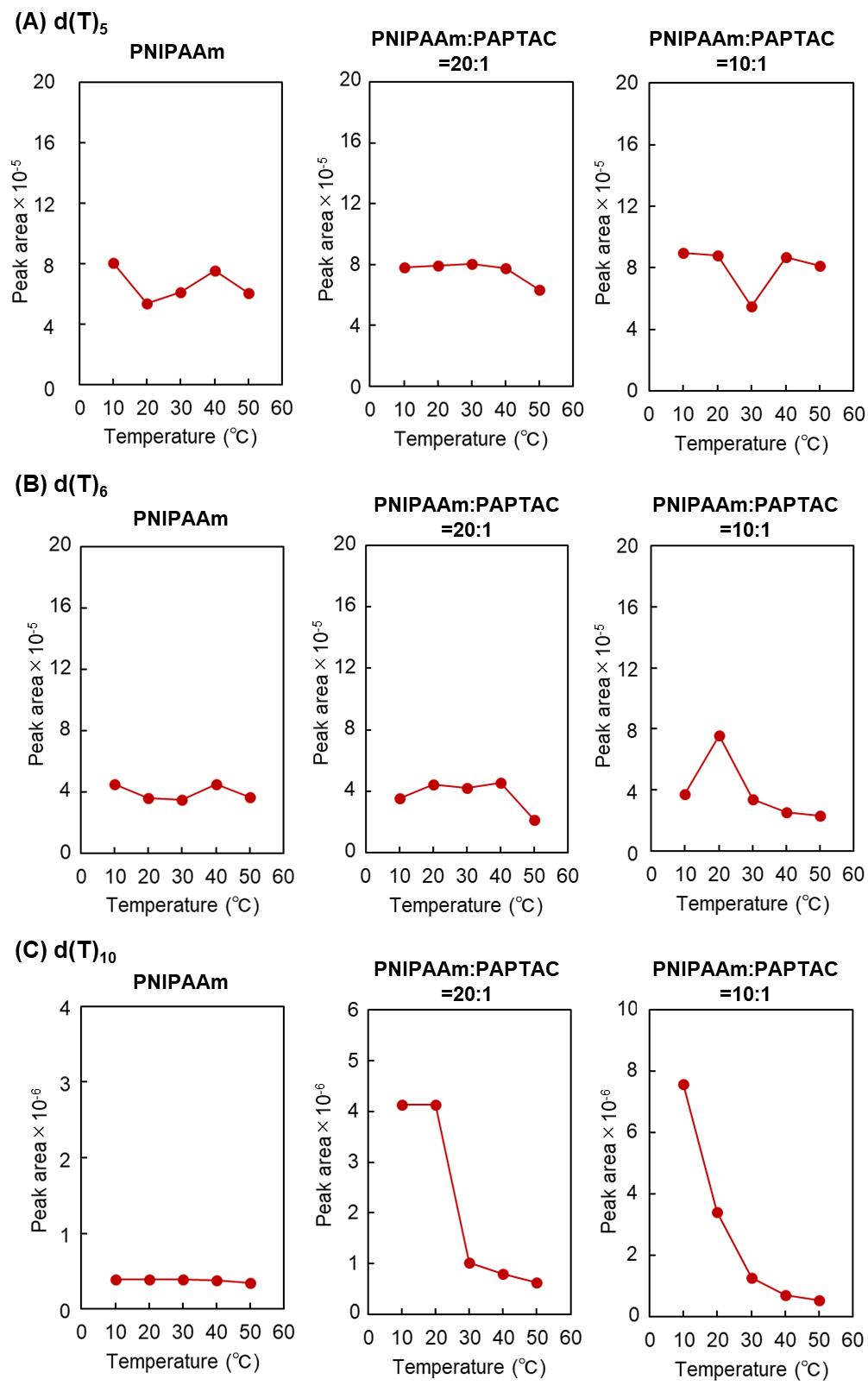


**Supplementary Fig. S6** Peak areas of oligonucleotides on the prepared mixed mode columns. (A) d(T)_5_ using 66.7 mM phosphate buffer solution (pH = 7.0), (B) d(T)_6_ using 66.7 mM phosphate buffer solution (pH = 7.0), and (C) d(T)_10_ using 66.7 mM phosphate buffer solution (pH = 7.0) + 100 mM NaCl. A flow rate of 1.0 mL/min was used. (Figures were drawn Microsoft PowerPoint 2019 Version 2112)
